# Testicular Expression of Antioxidant Enzymes and Changes in Response to a Slow-Release Deslorelin Implant (Suprelorin^®^ 4.7 mg) in the Dog

**DOI:** 10.3390/ani12182343

**Published:** 2022-09-08

**Authors:** Duygu Yaman Gram, Brigid Sexton, Narin Liman, Linda Müller, Murat Abay, Aykut Gram, Orsolya Balogh

**Affiliations:** 1 Department of Physiology, Faculty of Veterinary Medicine, Erciyes University, Kayseri 38280, Turkey; 2 Department of Small Animal Clinical Sciences, Virginia-Maryland College of Veterinary Medicine, Blacksburg, VA 24061, USA; 3 Department of Histology and Embryology, Faculty of Veterinary Medicine, Erciyes University, Kayseri 38280, Turkey; 4 Department of Obstetrics and Food Animal Medicine Clinic, University of Veterinary Medicine, 1078 Budapest, Hungary; 5 Department of Obstetrics and Gynecology, Faculty of Veterinary Medicine, Erciyes University, Kayseri 38280, Turkey

**Keywords:** canine, testis, infertility, antioxidants, deslorelin, GnRH superagonist, testicular downregulation, sperm, puberty

## Abstract

**Simple Summary:**

We investigated the expression of the testicular antioxidant enzyme system, i.e., superoxide dismutase (SOD1 and 2), catalase (CAT), glutathione peroxidase (GPx1), and glutathione disulfide reductase (GSR), in healthy adult and prepubertal dogs and after slow-release GnRH superagonist deslorelin (Suprelorin^®^ 4.7 mg subcutaneous implant, Virbac, France) treatment in adult dogs. qPCR and immunohistochemistry were used to detect gene expression and protein expression and localization, respectively. These antioxidant enzymes were variably expressed within cellular compartments in the seminiferous epithelium and the interstitium. SOD1, CAT, and GSR protein expression were also dependent on the stage of the seminiferous epithelium cycle in normal adult dogs. Deslorelin treatment and prepubertal status affected the expression and localization of the investigated antioxidant enzymes. Our results confirmed the presence of a functional antioxidant enzyme system in the canine testis, which was also observed during deslorelin-induced spermatogenic and steroidogenic arrest, as well as in prepubertal dogs. These results improve our current understanding of the local antioxidant protective mechanisms within the testis in the dog.

**Abstract:**

Spermatogenesis takes place in a hypoxic environment, and antioxidant enzymes protect germ and somatic cells from free radical-mediated damage. Expression of the antioxidant enzyme system in the canine testis has not yet been investigated. We hypothesized that the slow-release GnRH superagonist deslorelin 4.7 mg implant, which induces temporary reversible suppression of endocrine and germinative testicular function, would affect the testicular expression of antioxidant enzymes compared to untreated adult and prepubertal dogs. The goal of this study was to investigate and compare gene (by qPCR, in whole-tissue homogenates) and protein expression (by immunohistochemistry) of superoxide dismutase (SOD1, SOD2), catalase (CAT), glutathione peroxidase (GPx1), and glutathione disulfide reductase (GSR) in the testes of untreated adult (CON, *n* = 7), prepubertal (PRE, *n* = 8), and deslorelin-treated (DES, *n* = 5, 16 weeks after implantation) dogs. We found that in DES dogs, the gene expression of *SOD1* was significantly (*p* < 0.05) lower and *GPx1* was higher than in CON, and *SOD2* was higher than in PRE. Expression of all, except for the *SOD2* mRNA, differed between the CON and PRE dogs. Immunohistochemistry showed distinct cell-specific localization and expression patterns for the antioxidant enzymes in each experimental group. Additionally, in the CON animals, cell-specific SOD1, CAT, and GSR expression was dependent on the stage of the seminiferous epithelium cycle. These findings confirm that members of the antioxidant enzyme system are present in normal adult and prepubertal testis as well as in the deslorelin-treated downregulated adult canine testis, and that this local antioxidant system protects developing germ cells and somatic cells from oxidative damage. Different expression patterns of antioxidant enzymes in various germ cell populations and stages of the seminiferous epithelium cycle may indicate differences in their susceptibility to oxidative stress depending on their developmental and maturation stage. The continued presence of the antioxidant enzymes in the testis of DES dogs offers protection to spermatogonia as well as Sertoli and Leydig cells from oxidative stress during temporary infertility, potentially contributing to ensure the reversibility of suppression and the return of normal spermatogenesis and steroidogenesis after the end of deslorelin treatment.

## 1. Introduction

The subcutaneous implant containing the slow-release gonadotropin releasing hormone (GnRH) superagonist deslorelin (Suprelorin^®^ 4.7 mg or 9.4 mg, Virbac, France) is extensively and mainly used for the temporary, reversible suppression of spermatogenesis in male dogs as an alternative to surgical neutering [1,2,3]. Its mode of action is based on deslorelin’s increased stability and high binding affinity to GnRH receptors in the anterior pituitary, thereby preventing the stimulatory actions of endogenous GnRH on follicle stimulating hormone (FSH) and luteinizing hormone (LH) production [2,4]. In male gonads, LH and FSH control spermatogenesis by acting on their respective receptors in the Leydig and Sertoli cells, respectively, resulting in steroid hormone production, germ cell proliferation and differentiation during normal spermatogenesis [5,6,7]. GnRH superagonists initially mimic natural GnRH by stimulating gonadotropin synthesis and secretion, resulting in a “flare-up” effect, wherein testosterone concentration in male dogs is acutely increased for a few days to two weeks after deslorelin implantation, before steadily decreasing to undetectable levels [8,9,10,11,12]. After the short-lived “flare-up” period, prolonged exposure to GnRH superagonists will cause “desensitization” of pituitary gonadotrophs (by a post-receptor mechanism) and testicular desensitization to LH [3,4,13] through the downregulation of *LH receptor* expression [12]. Aspermia and complete sterility is usually observed within 2–12 weeks [8,10,14], as a result of spermatogenic arrest at the level of spermatogonia/primary spermatocytes and seminiferous tubule atrophy [15,16]. In dogs, the duration of spermatogenic arrest and testicular downregulation with the deslorelin implant has been shown to be dose-dependent [1,9,11]. In prepubertal males, a delay in the onset of puberty was observed [17].

While the broad clinical and endocrine consequences of slow-release GnRH superagonist treatments in male dogs are well-known, the local regulatory mechanisms and molecular changes within the testis are still not completely understood. Steroidogenic acute regulatory protein (STAR) has been identified as a key regulator of steroid hormone biosynthesis in several tissues [18]. The STAR protein has a role in the local modulation of testosterone production and is stimulated by LH, which increases steroidogenesis in Leydig cells [19,20]. GnRH superagonist treatment in male dogs resulted in decreased testicular STAR expression along with downregulation of other steroidogenic enzymes, all of which increased at the end of treatment, enabling the restart of steroidogenesis [21,22]. Cyclooxygenase 2 (COX-2/PTGS2) is thought to downregulate steroidogenesis and STAR activity in Leydig cells. Inhibition of COX-2 was found to increase the expression of STAR proteins in MA-10 mouse Leydig cells in vitro, resulting in increased steroidogenesis [23]. COX-2 was also found to be expressed in Sertoli and Leydig cells of dogs, and treatment with two different GnRH superagonist implants, azagly-nafarelin and buserelin acetate, affected COX-2 protein expression during downregulation and recrudescence of spermatogenesis [24]. The role of anti-Müllerian hormone (AMH) and insulin-like peptide 3 (INSL3) as biomarkers of Sertoli and Leydig cell function has recently been studied in the downregulated canine testis [12]. We found that, after deslorelin treatment, Sertoli and Leydig cells dedifferentiated and expressed higher levels of AMH and lower levels of INSL3 at the time of maximum downregulation, respectively, suggesting that these local mechanisms could further modulate steroidogenesis and spermatogenesis [12].

The testicular antioxidant system is an important defense mechanism which serves to protect somatic and germ cells from free radical-mediated damage. Reactive oxygen species (ROS) are normally produced in the testis during spermatogenesis and steroidogenesis as a byproduct of aerobic metabolism. At physiological concentrations, ROS contribute to various regulatory pathways during normal spermatogenesis and steroid hormone production, as well as to spermatozoa in acquiring fertilizing capacity [25,26,27,28]. When there is an imbalance between ROS production and elimination by the antioxidant defense system, oxidative stress ensues [29,30,31]. Oxidative stress results in pathological effects such as germ cell apoptosis, lipid peroxidation, protein oxidation, DNA and RNA oxidative damage, and has negative impacts on Leydig cell steroidogenesis, and can also disrupt the hypothalamo–pituitary–gonadal axis [25,26,32]. Because spermatogenesis occurs in a hypoxic environment, this process is especially vulnerable to oxidative stress [32]. Various exogenous and endogenous factors are implicated in increased generation of ROS and/or in decreased concentration or activity of the antioxidant defense mechanism, disrupting Sertoli and Leydig cell function and ultimately leading to infertility [25,26].

Enzymatic mechanisms are part of the antioxidant defense system against high ROS levels to protect cells from oxidative stress [25,30,33,34,35]. In the testis, this enzymatic defense system consists of superoxide dismutase (SOD), catalase (CAT), glutathione peroxidases (GPx), glutathione S-transferase (GST), and glutathione disulfide reductase (GSR) activities [32,36,37]. GPx, SOD1, SOD2, SOD3, and CAT mRNA and protein have been found in various cell types of mouse, rat, and Syrian hamster testis [38,39,40,41,42]. In rats, clear differences in antioxidant enzyme activities (SOD1 and 2, GPx, GST, and GSR) were dependent on the cell type, which indicates variability in the testicular cells in their response to oxidative stress [36,37]. High levels of ROS and/or low levels of antioxidant enzymes were correlated with infertility or subfertility in humans and animals in several studies [43,44,45,46,47]. In dogs, significantly lower seminal plasma SOD and CAT activities as well as plasma testosterone concentrations were found in asthenozoospermic compared to normal Beagle males [45]. Treatment with a slow-release deslorelin implant results in infertility, but the response of the testicular antioxidant enzyme system to this treatment is so far unknown. When a short-acting GnRH agonist buserelin was given to azoospermic dogs, blood testosterone and SOD levels in the testis were significantly increased at ten weeks after treatment compared to pretreatment values [44], although it is unclear whether the increased testicular SOD was a direct result of the upregulation of FSH and LH, or a consequence of increased testosterone production. It has previously been reported that GnRH superagonists induce changes in the canine testis, comparable to those observed in seasonally breeding animals out of the breeding season, such as the Syrian hamster or the roe deer [12,22,24]. The activity of testicular antioxidant enzymes in seasonal breeding animals varies by species as well as by breeding season. Testicular CAT protein expression decreased from a long day photoperiod (breeding season) to a short day photoperiod in the aged Syrian hamster [48]. In rams, increased SOD, CAT, and glutathione reductase (also known as glutathione disulfide reductase, GSR) activity was found in the seminal plasma during the nonbreeding season compared to the breeding season [49]. In dromedary camels, an increase in serum and epididymal fluid GPx levels was shown during the breeding season [50], while increased seminal plasma and vesicular gland fluid CAT, and decreased vesicular gland fluid SOD activity were found in wild boar/domestic pig hybrids during the autumn–winter compared to the spring–summer season [51].

The expression and localization of the antioxidant enzyme system in the canine testis has not yet been studied. We hypothesize that the testicular expression of SOD1, SOD2, CAT, GPx1, and GSR changes in response to treatment with a slow-release deslorelin implant, and that it is also dependent on the developmental stage of the canine testis when comparing sexually mature adult and prepubertal animals. Therefore, in this study, we investigated the expression and cellular localization of SOD1, SOD2, CAT, GPx1, and GSR in the testis of sexually mature adult dogs treated with the 4.7 mg deslorelin implant (Suprelorin^®^) and compared it to normal, healthy, untreated adult male dogs and to prepubertal dogs without established spermatogenesis. The findings of this study will increase our understanding of the implications of the antioxidant enzyme system in the canine testis and offer potential applications to study and understand canine male infertility.

## 2. Materials and Methods

### 2.1. Sample Collection

Twenty male dogs (various breeds and mixed breeds, 2 months–4.25 years, 4.1–44 kg) were divided into three experimental groups, i.e., untreated adult control (CON), untreated prepubertal (PRE), and adult deslorelin treated (DES) dogs. Testes samples were collected from seven healthy adult male dogs (CON group; mixed and various breeds, 1.25–4.25 years old, 12.4–44 kg) and from eight prepubertal male dogs (PRE group; 2–2.5 months, mixed breed, 4.1–6.3 kg), all undergoing routine neutering which was performed independent of the study. The DES group consisted of five adult, healthy, intact male beagles (2.5–3.75 years old, 10.9–13.9 kg) which were subcutaneously implanted (Day 0) with a 4.7 mg deslorelin implant (Suprelorin^®^, Virbac, France). At 16 weeks, these dogs were surgically castrated, and their testes were collected and preserved. On clinical examination, all participating animals were free of reproductive diseases and had two descended scrotal testes. All applicable national and institutional guidelines for the care and use of animals were followed. Animal procedures for the deslorelin-treated dogs were approved by IACUC (permitted by the Food Chain Safety and Animal Health Directorate of the Government Office for Pest County, permit number PEI/001/4557–4/2014).

The parenchyma of the testes were cut into small pieces and preserved in RNAlater™ (Thermo Fisher Scientific, Carlsbad, CA, USA) for 24 h at 4 °C, followed by storage at −80 °C for total RNA extraction. For immunohistochemistry, testis samples were fixed in 10% neutral phosphate-buffered formalin for 24 h at 4 °C, washed daily in phosphate buffered saline for one week, dehydrated in graded ethanol series, and embedded in paraffin.

### 2.2. Isolation of Total RNA, cDNA Synthesis, and Reverse Transcription

Testis tissue samples preserved in RNAlater™ were homogenized in a TissueLyser LT (Qiagen, Germantown, MD, USA). Isolation of total RNA was performed using TRIzol^TM^ reagent (Thermo Fisher Scientific, Carlsbad, CA, USA), according to the manufacturer’s instructions. Quantitation and quality of extracted total RNA were evaluated using a NanoDrop^TM^ One spectrophotometer (Thermo Fisher Scientific). To remove genomic DNA contamination, all total RNA samples were treated with RQ1 RNase-Free DNase (Promega, Madison, WI, USA) according to the manufacturer’s instructions. Reverse transcription was performed using RT reagents from Applied Biosystems (Carlsbad, CA, USA) with random hexamers as primers, following the manufacturer’s recommendations and with modifications according to a previously published protocol [52]. Samples (each containing 200 ng DNAse-treated total RNA) were run in an Eppendorf MasterCycler Pro as follows: 8 min at 21 °C, followed by 15 min at 45 °C, and then 5 min at 99 °C to stop the reaction.

### 2.3. Semi-Quantitative RT-PCR 

Semi-quantitative real-time (TaqMan) PCR was performed using the ABI PRISM 7500 Fast Sequence Detection System (Applied Biosystems). The 25 μL reaction mixture contained 200 nM TaqMan Probe, 300 nM of each primer, 12.5 μL Fast Start Universal Probe Master (ROX) (Roche Diagnostics GmbH, Mannheim, Germany), and 5 μL of cDNA corresponding to 200 ng total RNA. All samples were run in duplicates in 96-well optical plates (Applied Biosystems). The amplification conditions were: initial denaturation at 95 °C for 10 min followed by 40 cycles for 15 s at 95 °C and 60 s at 60 °C. Canine-specific *glyceraldehyde-3-phosphate dehydrogenase (GAPDH)* and canine *peptidylprolyl isomerase A (PPIA*/*cyclophyllin A)* were used as reference genes, similarly to our previous study [12]. The *GAPDH* primers and TaqMan probe sequences used here had been previously described [52] and were synthesized by Integrated DNA Technologies (Coralville, IA, USA). The TaqMan probe for *GAPDH* was labeled at the 5′ end with 6-carboxyfluorescein (FAM) as the reporter dye and at the 3′ end with 6-carboxytetramethyl-rhodamine (TAMRA) as the quencher. Commercially available canine-specific TaqMan Gene Expression Assays containing primers and probe mixture for *cyclophyllin A*, *SOD1*, *SOD2*, *CAT*, *GPx1*, and *GSR* (Applied Biosystems) were used. A list of primers and probes used is presented in Table 1. As negative controls, the so-called RT minus control (DNAse treated samples from reverse transcription reaction without reverse transcriptase) as well as nontemplate controls (samples with sterile water instead of cDNA) were used in order to screen for DNA contamination. The comparative CT (ΔΔCT) method was used to calculate relative gene expression (RGE) according to the manufacturer’s instructions of the ABI PRISM 7500 system and as described previously [12,52,53,54] using both reference genes. The sample with the lowest detectable amount of transcript was used as the calibrator.

### 2.4. Immunohistochemistry (IHC)

Localization of antioxidant enzymes, i.e., SOD1, SOD2, CAT, GPx1, and GSR, was detected in testicular samples using a previously published standard immunoperoxidase method [55]. Briefly, 2–3 μm thick tissue slides were prepared from paraffin-embedded testicular tissues. Sections were then mounted on SuperFrost microscope slides (Menzel-Glaeser, Braunschweig, Germany). Deparaffinization of the slides was achieved in xylene, and slides were rehydrated in a graded sequence of ethanol. Epitope/antigen retrieval was performed by heating the deparaffinized slides in a microwave oven at 600 W for 15 min in a 10 mM citrate buffer (pH6.0). To prevent false positive detection and high background staining, endogenous peroxidase activity was blocked using 0.3% hydrogen peroxide in methanol. For blocking nonspecific binding of antibodies, sections were incubated with a blocking solution (Ultra V Block, Thermo Fisher Scientific, LabVision Corporation, Fremont, CA, USA) for 5 min. Tissue sections were then incubated with the primary antibodies (Table 2) overnight at 4 °C. All antibodies were diluted in an antibody diluent solution (Invitrogen Corporation, 00-3118, Carlsbad, CA, USA). As negative controls, samples incubated with nonimmunized IgGs (isotype controls) of the same species and at the same protein concentration as the primary antibody, as well as without the primary antibodies (negative control), were used. The next day, following rinsing of samples with PBS (0.8 mM Na2HPO4, 1.47 mM KH2PO4, 2.68 mM KCl, 137 mM NaCl; pH 7.2–7.4), slides were incubated with secondary antibodies, i.e., UltraTek HRP anti-Mouse antibody (purchased from ScyTek Laboratories Inc., Logan, UT, USA). Signal intensity was enhanced by incubating tissue samples with streptavidin peroxidase (Ready-To-Use, Thermo Fisher Scientific, TS-125-HR) for 20 min at room temperature. Peroxidase activity was achieved by using DAB plus substrate system (Thermo Scientific, Lab Vision, TA-125-HDX). Slides were counterstained with hematoxylin and, following dehydration in a graded ethanol series followed by xylene, they were mounted in Entellan (Entellan^®^ new, Merck, 1079610500, Darmstadt, Germany).

### 2.5. Expression of Antioxidant Enzymes in the Testes of Adult CON Group Dogs in Different Seminiferous Epithelium Cycle Stages

We categorized the stages of the canine seminiferous epithelium into eight stages based on the acrosome system and morphology of the developing spermatid nucleus, as described previously [56]. Protein expression of the antioxidant enzymes was evaluated according to the seminiferous epithelium cycle stage.

### 2.6. Statistical Analysis

One-way ANOVA followed by Tukey Honestly Significant Difference or a Kruskal–Wallis test followed by Bonferroni correction for multiple comparisons was used on logarithmically transformed data to compare RGE between the treatment groups using IBM^®^ SPSS^®^ Statistics version 27 (Armonk, NY, USA). RGE of each target gene was included as the dependent variable and each treatment group (CON, DES, and PRE) as the fixed effect. Results from all analyses on RGE are presented as geometric mean (Xg) ± deviation factor (DF). A *p* value of <0.05 was considered significant.

## 3. Results

### 3.1. SOD1 and SOD2

**Figure 1:**
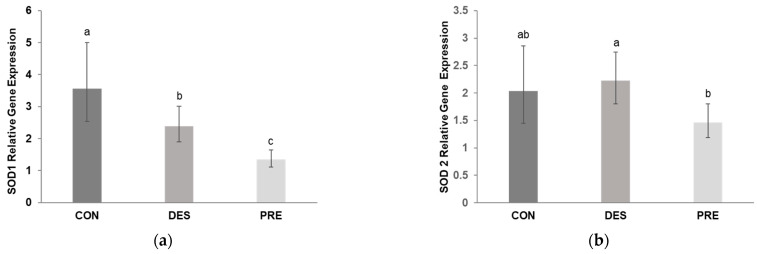
Relative gene expression of superoxide dismutase 1 and 2 (*SOD1* and *SOD2*) in the canine testis: (**a**) Relative gene expression of *SOD1* was significantly different between the untreated adult dogs (CON), deslorelin treated adult dogs (DES), and prepubertal dogs (PRE) (*p* ≤ 0.045); (**b**) Relative gene expression of *SOD2* differed only between the DES and PRE dogs (*p* = 0.034). Bars show the geometric mean and whiskers show the deviation factor. Different letters indicate significant differences between experimental groups (*p* < 0.05).

Deslorelin treatment had a substantial effect on testicular gene expression of *SOD1*. We found that *SOD1* mRNA levels in the testis were significantly lower in DES compared to CON dogs (*p* = 0.045) but were higher than in PRE animals (*p* = 0.004). In the PRE dogs, testicular *SOD1* gene expression was lower than in CON (*p* < 0.0001; Figure 1a). On the other hand, *SOD2* gene expression did not differ between the DES and CON animals (*p* = 0.852), while PRE dogs had lower mRNA levels than DES (*p* = 0.034) but were not different from CON (*p* = 0.065) (Figure 1b).

**Figure 2:**
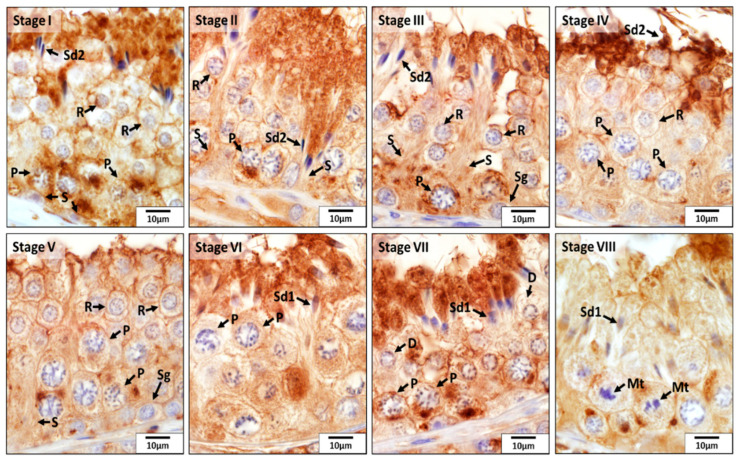
Localization of superoxide dismutase 1 (SOD1) during different stages (**I**–**VIII**) of the seminiferous epithelium cycle in the testes of untreated adult dogs (CON). In all stages of the seminiferous epithelium, immunostaining for SOD1 was observed in Sertoli cells (S) and in germ cells, i.e., spermatogonia (Sg), diplotene (D) and pachytene (P) spermatocytes, round spermatids (R), elongating (Sd1) and elongated spermatids (Sd2). In addition, meiotic spermatocytes (Mt) also showed a positive reaction for SOD1.

According to the morphological analysis of the seminiferous tubules in CON dogs, immunohistochemistry staining of SOD1 in all germ cells—including spermatogonia; primary spermatocytes (zygotene, pachytene, and diplotene spermatocytes); and round, elongating, and elongated spermatids—was mainly present in the seminiferous tubules at stages I through VIII. Especially, the expression of SOD1 in the adluminal compartment of the seminiferous epithelium was stronger than that in the basal compartment during stages I to IV and VI to VIII, which was due to the presence of stronger immunoreactivity in the elongating and elongated spermatids. However, this was not the case for stage V, in which only round spermatids were available. Moreover, in contrast to other stages, in stage VIII, elongating and elongated spermatids were stained weakly for SOD1 (Figure 2).

**Figure 3:**
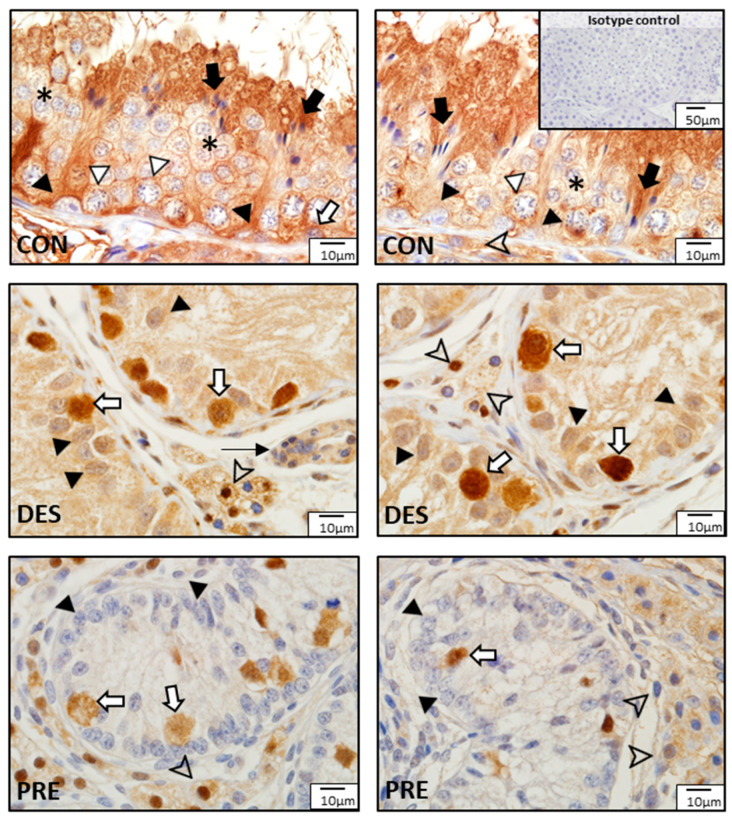
Detection of superoxide dismutase 1 (SOD1) protein by immunohistochemistry in the canine testis according to experimental groups: SOD1 in the untreated adult dogs (CON) is visible in all germ cells, including spermatogonia (white arrow), spermatocytes (white triangle), round spermatids (asterisks), and elongating and elongated spermatids (black arrows), as well as in Sertoli cells (black triangles) and Leydig cells (arrowhead). In deslorelin treated adult dogs (DES), spermatogonia appeared with strong immunopositivity (white arrows), while Sertoli cells (black triangle) and Leydig cells (arrowheads) had slightly weaker SOD1 staining. In the prepubertal dogs (PRE), gonocytes (white arrows) and Leydig cells (arrowheads) were immunopositive, while Sertoli cells sporadically stained weakly (black triangles). Staining in blood vessels was also present (thin black arrow).

**Figure 4:**
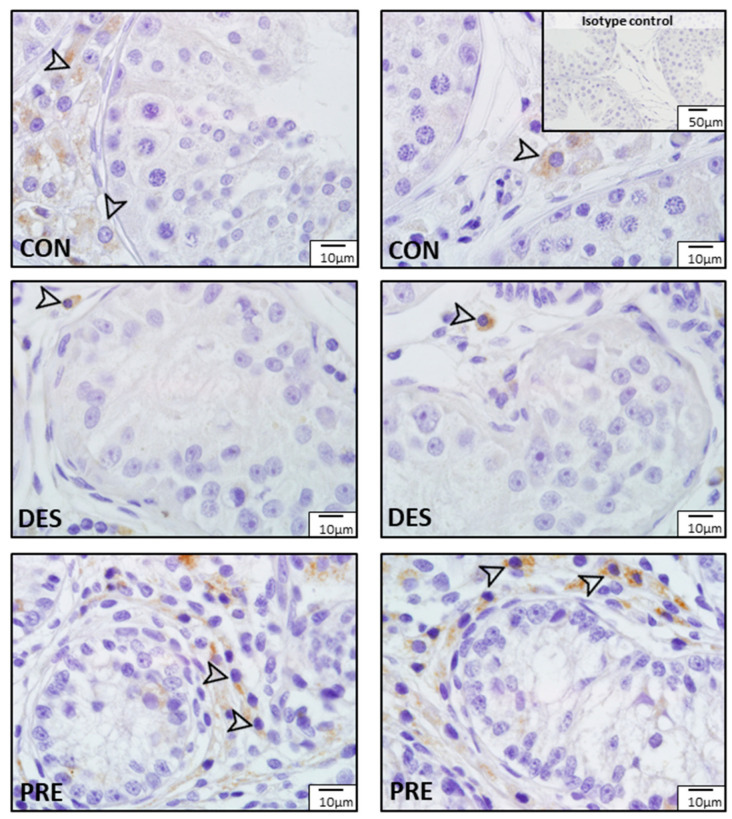
Detection of superoxide dismutase 2 (SOD2) protein by immunohistochemistry in the canine testis according to experimental groups: No or sporadic weak signals for SOD2 are visible in the Leydig cells (arrowheads) of the untreated adult dogs (CON), the deslorelin treated adult (DES), and in the prepubertal (PRE) dogs.

Immunostaining for SOD1 and SOD2 was different among experimental groups (Figure 3 and Figure 4). In the CON dogs, SOD1 was localized in Sertoli cells and in all germ cell types of the seminiferous epithelium, and also in the interstitial Leydig cells. The strongest immunostaining appeared in spermatids and in Sertoli cells (Figure 3). In the DES animals, the strongest immunostaining appeared in spermatogonia, while Sertoli and Leydig cells also showed immunopositivity. In the testes of PRE dogs, gonocytes and Leydig cells showed similar staining intensity, while Sertoli cells seemed to have either no or only sporadic, weak staining. SOD1 protein was also detected in blood vessels in all experimental groups (Figure 3). As for SOD2, which was present exclusively in the Leydig cells, either weak signals or no signals were visible in all experimental groups (Figure 4).

### 3.2. Catalase (CAT)

**Figure 5:**
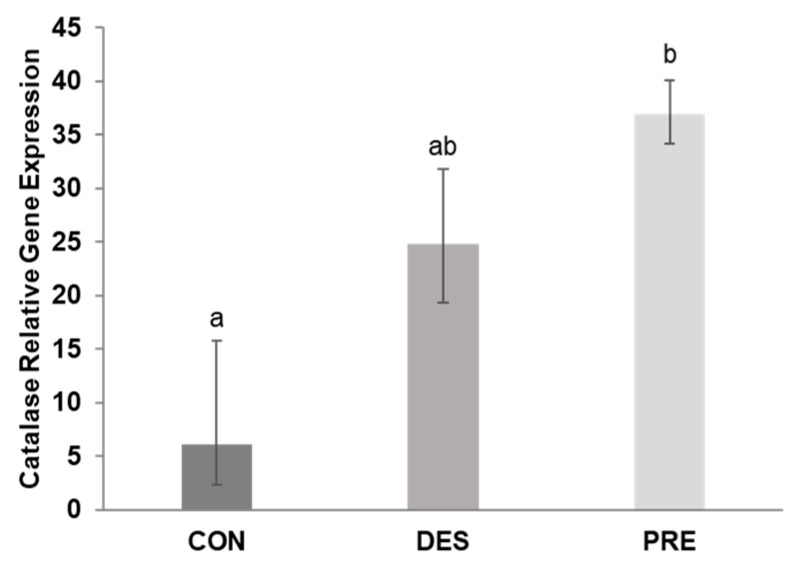
Relative gene expression of catalase (*CAT)* in the canine testis: *CAT* mRNA levels differed significantly between the untreated adult (CON) and prepubertal dogs (PRE) (*p* < 0.0001), while the deslorelin treated adult dogs (DES) were intermediate (*p* ≥ 0.156). Bars show the geometric mean and whiskers the deviation factor. Different letters above bars indicate significant differences between experimental groups (*p* < 0.05).

*CAT* gene expression in the testis was not affected by deslorelin treatment compared to CON dogs (*p* = 0.156). *CAT* mRNA levels in the testes of PRE dogs were higher than in CON (*p* < 0.0001) but were not different from the DES dogs (*p* = 0.320) (Figure 5).

**Figure 6:**
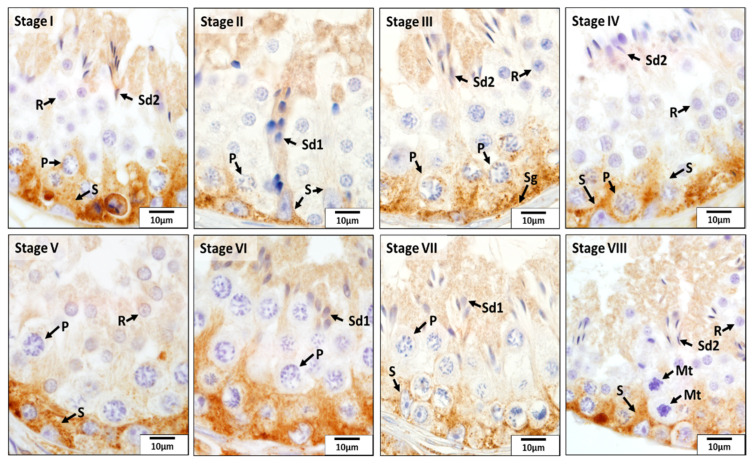
Localization of catalase (CAT) during different stages (**I**–**VIII**) of the seminiferous epithelium cycle in the testes of untreated adult dogs (CON). In all eight stages of the seminiferous epithelium cycle, immunostaining for CAT was observed in the Sertoli cells (S), spermatogonia (Sg), pachytene (P) spermatocytes, round spermatids (R), and elongating (Sd1) and elongated spermatids (Sd2). Meiotic spermatocytes (Mt) showed no signals for CAT.

As for CAT, this enzyme was mainly localized in Sertoli cells, spermatogonia, pachytene spermatocytes, and elongating and elongated spermatids in all eight stages of the seminiferous epithelium cycle of the CON dogs (Figure 6). Especially, its expression in the basal compartment of the seminiferous epithelium was stronger than that in the adluminal compartment during stages IV and V, which was due to the stronger immunoreactivity present in Sertoli cells, spermatogonia, and pachytene spermatocytes. However, in other stages of the seminiferous epithelium cycle, elongating and elongated spermatids were also stained strongly for CAT. No or weak signals were detected for CAT in other germ cells.

**Figure 7:**
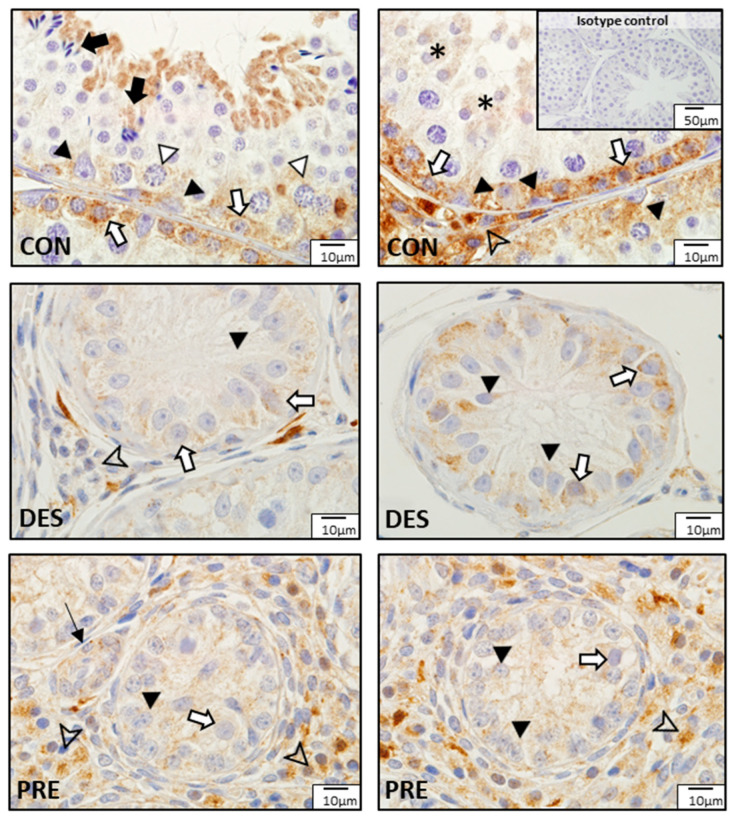
Detection of catalase (CAT) proteins by immunohistochemistry in the canine testis according to experimental groups: CAT in the untreated adult dogs (CON) is visible in the spermatogonia (white arrows), spermatocytes (white triangles), round spermatids (asterisks), and elongating and elongated spermatids (black arrows). Sertoli cells (black triangles) also stained and Leydig cells (arrowhead) were strongly immunopositive. In deslorelin treated adult dogs (DES), weak signals appeared in spermatogonia (white arrows) and in Sertoli cells (black triangles) localized in the cytoplasm, close to the basement membrane; Leydig cells (arrowhead) stained inconsistently. In the prepubertal dogs (PRE), Sertoli cells (black triangles) and gonocytes (white arrows) weakly stained, while Leydig cells (arrowheads) stained more strongly. Blood vessels (thin black arrow) stained in all experimental groups.

Comparison of CAT protein expression among experimental groups showed different localization patterns (Figure 7). In CON dogs, besides the seminiferous epithelium mentioned above, strong CAT immunostaining was also visible in Leydig cells. In the DES dogs, weak positive signals for CAT were found in spermatogonia and Sertoli cells, while Leydig cells had no or sporadic weak immunostaining. In the testes of PRE dogs, weak immunostaining was visible within the seminiferous tubules in Sertoli cells and gonocytes, while Leydig cells in the interstitium appeared to have relatively stronger CAT immunosignals. Blood vessels also stained in all experimental groups.

### 3.3. GPx1 and GSR

**Figure 8:**
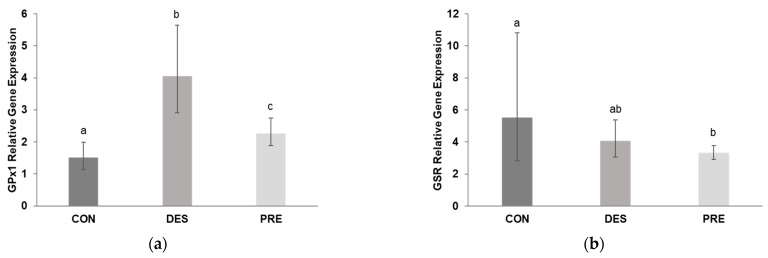
Relative gene expression of glutathione peroxidase (*GPx1*) and glutathione disulfide reductase (*GSR*) in the canine testis: (**a**) *GPx1* mRNA levels were significantly different between the untreated adult dogs (CON), deslorelin treated adult dogs (DES), and prepubertal dogs (PRE). *GPx1* gene expression in the testis of CON dogs was significantly lower than in DES (*p* < 0.0001) and in PRE (*p* = 0.017), and was higher in DES than in PRE animals (*p* = 0.003); (**b**) Gene expression of *GSR* differed only between the CON and PRE dogs (*p* = 0.028). Bars show the geometric mean and whiskers the deviation factor. Different letters above bars indicate significant differences between experimental groups (*p* < 0.05).

Testicular *GPx1* gene expression (Figure 8a) was significantly higher in DES compared to CON (*p* < 0.0001) and PRE dogs (*p* = 0.003); *GPx1* mRNA levels were also higher in PRE than in CON animals (*p* = 0.017). Deslorelin treatment did not affect *GSR* gene expression (*p* = 0.705 for CON vs. DES), while CON dogs had higher *GSR* gene expression than PRE animals (*p* = 0.028) (Figure 8b).

**Figure 9:**
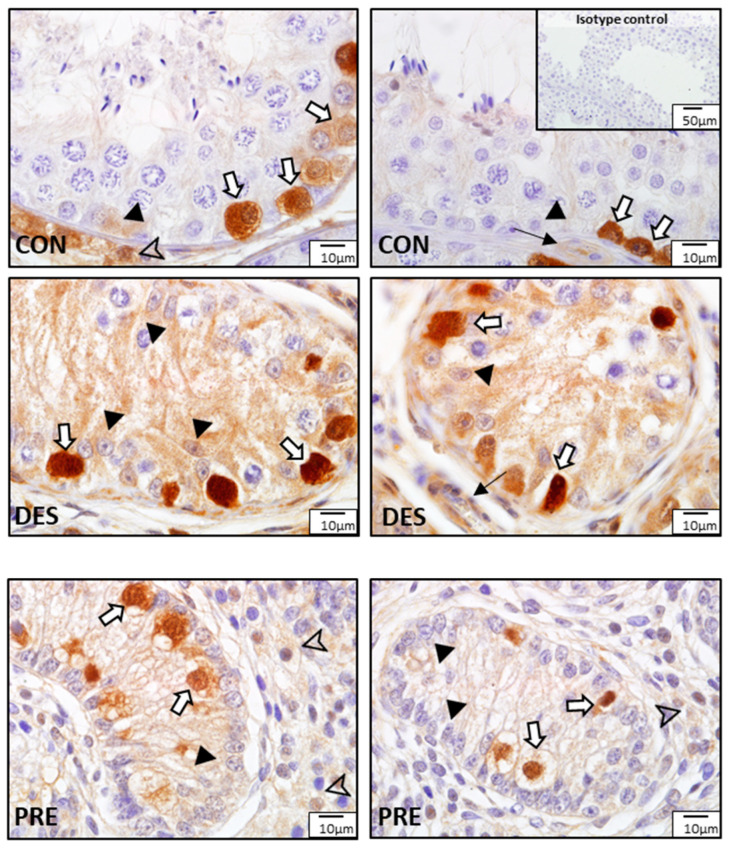
Detection of glutathione peroxidase (GPx1) by immunohistochemistry in the canine testis according to experimental groups: GPx1 in the untreated adult dogs (CON) is visible in spermatogonia (white arrows), Leydig cells (arrowhead), Sertoli cells (black triangles), and blood vessels (thin black arrow). In deslorelin treated adult dogs (DES), spermatogonia (white arrows) were strongly immunopositive, and Sertoli cells (black triangles) and blood vessels (think black arrow) also stained. In the prepubertal dogs (PRE), signals are visible in gonocytes (white arrows), Sertoli cells (black triangles), and Leydig cells (arrowheads).

GPx1 protein expression in the testes of CON, DES, and PRE dogs is shown in Figure 9. In the CON animals, GPx1 protein was localized mainly in spermatogonia and Leydig cells, while all other germ cells were devoid of signals. Sertoli cells showed weak sporadic staining. In DES dogs, spermatogonia appeared to have strong GPx1 immunostaining, and Leydig cells also stained. Sertoli cells seemed to have stronger GPx1 signals in the DES than in the CON dogs. In the PRE testis, strong GPx1 protein signals were detected in gonocytes, while Sertoli and Leydig cells stained weakly. Signals for GPx1 in blood vessels were also visible in all experimental groups.

**Figure 10:**
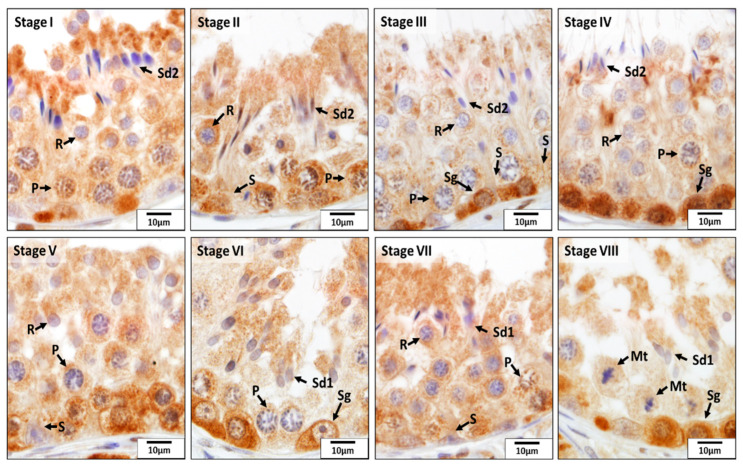
Localization of glutathione disulfide reductase (GSR) during different stages (**I**–**VIII**) of the seminiferous epithelium cycle in the testes of untreated adult dogs (CON). In all eight stages of the seminiferous epithelium cycle, immunostaining for GSR was observed in Sertoli cells (S) and in the germ cells, including spermatogonia (Sg); pachytene (P) spermatocytes; round (R), elongating (Sd1), and elongated spermatids (Sd2);. In addition, the meiotic spermatocytes (Mt) showed a positive reaction for GSR.

Similar to SOD1, at various stages of the seminiferous epithelium cycle of the CON dogs, GSR protein was detected in spermatogonia; primary spermatocytes (zygotene, pachytene, and diplotene spermatocytes); and round, elongating, and elongated spermatids. Its expression in the basal compartment and adluminal compartment during stages I, II, and V to VII was highest, but it diminished greatly in the adluminal compartment at stages III, IV, and VIII (Figure 10).

**Figure 11:**
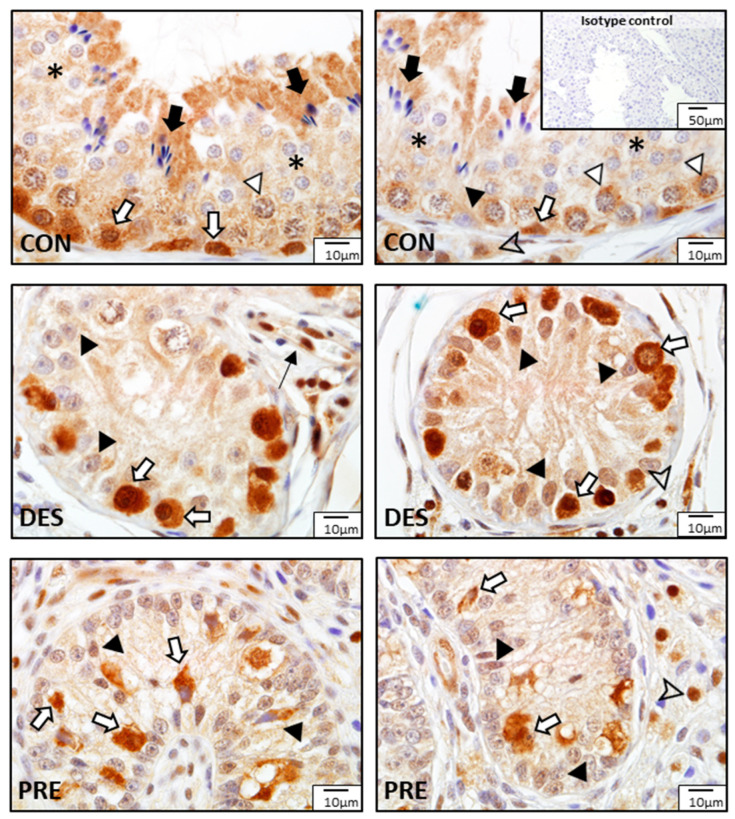
Detection of glutathione disulfide reductase (GSR) proteins by immunohistochemistry in the canine testis according to experimental groups: GSR stained strongly in the spermatogonia (white arrows) of the untreated adult dogs (CON), as well as in the spermatocytes (white triangles), round spermatids (asterisks), elongating and elongated spermatids (black arrows), Sertoli cells (black triangle), and Leydig cells (arrowhead). In the deslorelin treated dogs (DES), immunosignals are visible in spermatogonia (white arrows), Sertoli cells (black triangles), Leydig cells (arrowhead), and blood vessels (thin black arrow). In the prepubertal dogs (PRE), gonocytes (white arrows), Sertoli cells (black triangles), and Leydig cells (arrowhead) were immunopositive.

GSR protein expression in dogs in the different experimental groups is shown in Figure 11. In the CON dogs, GSR was localized to all germ cells with strongest immunostaining in spermatogonia, spermatocytes, and elongating spermatids. The strongest GSR signals were visible in the spermatogonia and gonocytes of the DES and PRE dogs, respectively. Leydig cells, Sertoli cells, as well as blood vessels (endothelial cells and media) also showed positive immunostaining in all experimental groups.

## 4. Discussion

Spermatogenesis is an active process resulting in the development of haploid spermatozoa from diploid spermatogonia. During this extremely active process, high mitochondrial oxygen consumption by germ cells and poor testicular vascularization causes a hypoxic environment [32,57]. Since germ cells and somatic cells (e.g., Leydig and Sertoli cells) are sensitive to oxidative stress, locally produced antioxidant enzymes protect the testis from free radical-mediated damage, which is important for normal physiology of the male gonad. The expression of antioxidant enzymes in the testis has been previously described in several species [36,37,38,39,40,41,42] but not in the dog. In this study, we found that in normal adult dogs, among the five antioxidant enzymes investigated, SOD1, CAT, and GSR were expressed in all germ cells with clear differences in their expression patterns among the developing germ cells. Differentiating sperm cells appeared to have the strongest SOD1, CAT, and GSR expressions, while spermatogonia had strong immunoreactivity for CAT, GPx1, and GSR. These findings support previous studies describing different distribution patterns of antioxidant enzymes among germ cell populations [36,37,38] and thus their varying susceptibility to free radical-mediated damage. Normal adult canine Sertoli and Leydig cells also stained for all antioxidant enzymes; however, the strongest protein expressions in Sertoli cells seemed to be present for SOD1 and CAT, and in Leydig cells for SOD1, CAT, and GPx1. In contrast to rats [38], SOD2 protein in dogs was only localized to Leydig cells. As ROS have an important role in normal steroidogenic activity in the testis through Leydig cells (as shown in other species) [25,28], it is not surprising that also in dogs, Leydig and Sertoli cells are equipped with an antioxidant enzyme system to maintain an appropriate redox state.

An interesting and novel finding was that cell-specific expression of SOD1, CAT, and GSR in the canine testis also depended on the cycle of the seminiferous epithelium. This may be an indication that, as germ cells go through the steps of spermatogenesis, their susceptibility to oxidative stress—and hence their need for the provision of specific antioxidant enzymes—changes. Furthermore, as physiological levels of ROS are required for normal spermatogenesis (including the potential for spermatogonial stem cell renewal) [25,58], the differential antioxidant protection likely helps cells within the seminiferous tubules to establish the delicate balance of ROS levels for normal spermatogenesis to proceed.

A balanced redox state through the antioxidant system is crucial for maintaining normal steroidogenesis and spermatogenesis [25,32]. Sex steroids and gonadotropins play a role in controlling the testicular antioxidant system. Therefore, the administration of exogenous androgens or estrogens, and both hypo- and hypergonadotropism, result in reduced testicular antioxidant enzyme activities, disruption of spermatogenesis, and germ cell apoptosis [32]. Accordingly, in rats, short-term testosterone treatment for eight days decreased serum LH levels, testicular lipid peroxidation, and enzyme activities of CAT, GPx, and GST, without affecting the testicular SOD activities and serum testosterone levels or testis weight [59]. Androgen deficiency caused by ethane dimethane sulfonate administration decreased the mRNA expression of GPx and CAT, and increased SOD expression in the rat testis [60]. Short-term GnRH antagonist treatment in rats, which resulted in decreased serum testosterone levels but not in testicular atrophy, also decreased testicular CAT and GPx activities without affecting lipid peroxidation or SOD activities [59]. In dogs, the application of a slow-release deslorelin implant causes “desensitization” of pituitary GnRH receptors, thereby reducing the release of gonadotropins and gonadal steroid hormone production [2,4,8,9,11,12]. This decrease in LH concentrations in peripheral blood, as well as in the intratesticular testosterone production, may result in the suppression of the testicular antioxidant system, which could lead to the production of free radicals, and consequently, germ cell apoptosis and disrupted spermatogenesis. Dogs injected with the GnRH analogue, buserelin acetate, first had increased testicular SOD levels during the flare-up period [44]; the authors speculated that after the flare-up, testicular SOD may decrease, similar to testosterone. We may hypothesize that deslorelin treatment will suppress the antioxidant enzyme system, which will ultimately contribute to spermatogenic arrest. However, the initial changes during early disruption of spermatogenesis cannot be proven in our study, as we studied the testis of DES dogs at the time of maximum downregulation of spermatogenesis (16 weeks postimplantation), when developing germ cells were not detected anymore. Furthermore, we found different expression patterns for the various antioxidant enzymes in the DES dogs. The gene expression of *SOD1* was downregulated while *GPx1* was upregulated in whole testicular tissue homogenates of DES dogs compared to CON. Because deslorelin treatment arrests spermatogenesis at the level of spermatogonia/primary spermatocytes in dogs [15,16], the expression of the antioxidant enzymes localized primarily in the haploid germ cells at later stages of development in the seminiferous tubules of untreated adult dogs would not be expressed in deslorelin-treated dogs. This may explain the decreased gene expression of *SOD1* in the DES compared to the CON animals. Regarding GPx1, spermatogonia showed strong immunoreactivity both in the DES and CON dogs. In contrast, in the downregulated testis, Sertoli cell seemed to more strongly express GPx1 protein compared to the testis of CON animals, which likely explains the higher mRNA expression levels found in the DES dogs. Within the seminiferous tubules of the DES animals, spermatogonia and Sertoli cells continued to strongly express not only GPx1 but also GSR and SOD1 proteins. Therefore, these antioxidant enzymes seem to be important in the protection of these cells during periods of testicular inactivity in the dog. Some of the antioxidant enzymes in CON dogs were strongly expressed in developing germ cells, e.g., SOD1, GSR, and CAT; hence the changes in the ratio of the antioxidant system could have contributed to the increased production of ROS, inducing germ cell apoptosis and the disruption of spermatogenesis in the DES dogs. However, this may have happened earlier, within a shorter period of time after deslorelin implant insertion, when the developing germ cells were still present in the testis.

The status of the testis in the GnRH superagonist-induced infertile male dog resembles the status of the testis of the inactive seasonal breeding animal [12,22,24]. Several studies have noted differences in reproductive fluid or testicular antioxidant enzyme activity or levels between the breeding and nonbreeding periods of seasonally breeding animals [48,50,51,61]. In the Syrian hamster, short day photoperiods resulted in decreased testicular CAT protein expression along with decreased lipid peroxidation [48]. Seminal plasma CAT of wild boar/domestic pig hybrids was found to be increased during the breeding season [59], while in rams, either no difference or decreased seminal plasma GPx activity was reported between the breeding and the nonbreeding season or the equatorial photoperiod, respectively [49,61]. Testicular expression was not examined in these studies and thus cannot be compared to our results.

Compared to the CON dogs, we found significantly decreased *SOD1* and *GSR* in the PRE dogs, which may be explained by the lack of developing germ cells which strongly express these proteins in normal adult dogs. On the other hand, the increased *CAT* and *GPx1* gene expression in PRE dogs may be due to the seemingly stronger protein expression in Sertoli cells and the different volume ratio of seminiferous tubules and interstitium between the testes of PRE and CON animals. For most of our genes of interest (*SOD1, SOD2,* and *GPx1*), their mRNA expression levels in DES dogs were significantly different from those in PRE dogs. This supports observations from previous studies describing differences in some gene and protein expression patterns between the testes of prepubertal and GnRH superagonist-treated dogs [12,22,24,62], pinpointing the contrast between a developmental lack of established germinal and endocrine testicular function and pharmacologically induced infertility in adult dogs, also at the molecular level.

## 5. Conclusions

In this study we described the antioxidant enzyme system in the testis of normal adult, prepubertal, and deslorelin-treated adult dogs. Our results show that there was a distinct cell-specific localization and expression pattern for each of the antioxidant enzymes studied, i.e., SOD1, SOD2, CAT, GPx1, and GSR, offering protection to various germ and somatic cell populations depending on the developmental stage of the testis, i.e., adult vs. prepubertal, as well as following cessation of spermatogenesis and steroidogenesis during induced infertility with a slow-release deslorelin implant. Furthermore, in case of the SOD1, CAT, and GSR proteins, their cell-specific expression patterns were also dependent on the stage of the seminiferous epithelium cycle in normal adult dogs. Different expression patterns of these antioxidant enzymes in various germ cell populations and stages of the seminiferous epithelium cycle may indicate differences in their susceptibility to oxidative stress, depending on their developmental and maturation stage. These findings suggest that the continued presence of an antioxidant enzyme system during GnRH superagonist-induced testicular downregulation may still serve to protect spermatogonia as well as Sertoli and Leydig cells from free radical-mediated damage during temporary infertility, perhaps contributing to the reversibility of testicular suppression and the return of normal spermatogenesis and steroid hormone production after cessation of deslorelin’s effects. Overall, the results of this study have increased our knowledge of the implications of the antioxidant enzyme system in the canine testis and offer a basis for further studies on canine testicular physiology and male infertility.

## Figures and Tables

**Table 1:** Primer and TaqMan probe sequences or assay IDs (if commercial products) for each target gene with corresponding amplified amplicon length and accession numbers.

Target Gene	Primer and Probe Sequences or Assay ID	Amplicon Length	Accession Number
*GAPDH*	Forward: 5′-GCT GCC AAA TAT GAC ATC A-3′ Reverse: 5′-GTA GCC CAG GAT GCC TTT GAG-3′ TaqMan probe: 5′-TCC CTC CGA TGC CTG CTT CAC TAC CTT-3′	75 bp	AB028142.1
*Cyclophyllin A*	Cf03986523_gH	92 bp	XM_843327.1
*SOD 1*	Cf02624276_m1	111 bp	NM_001003035.1
*SOD 2*	Cf02640459_g1	96 bp	XM_533463.7
*CAT*	Cf02621930_g1	109 bp	NM_001002984.1
*GPx1*	Cf02731517_s1	81 bp	NM_001115119.1
*GSR*	Cf02639488_m1	94 bp	XM_014120078.3

GAPDH: glyceraldehyde-3-phosphate dehydrogenase (as sequence previously published [52]); SOD 1, 2: superoxide dismutase 1 and 2; CAT: catalase; GPx1: glutathione peroxidase 1; GSR: glutathione disulfide reductase.

**Table 2:** Antibodies used for immunohistochemistry for the detection of superoxide dismutase 1 and 2 (SOD1, SOD2), catalase (CAT), glutathione peroxidase 1 (GPx1), and glutathione disulfide reductase (GSR) in canine testicular tissues.

Antigen	Company	Catalog Number	Species/Type	Dilution
SOD 1	Santa Cruz Biotechnology	sc-101523	Mouse monoclonal	1:200
SOD 2	Santa Cruz Biotechnology	sc-137254	Mouse monoclonal	1:100
CAT	Santa Cruz Biotechnology	sc-271803	Mouse monoclonal	1:100
GPx1	Santa Cruz Biotechnology	sc-133160	Mouse monoclonal	1:100
GSR	Santa Cruz Biotechnology	sc-133245	Mouse monoclonal	1:100

## Data Availability

The data presented in this study belong to the authors and are available on reasonable request from the corresponding authors.
